# Patient-Oriented In Vitro Studies in Duchenne Muscular Dystrophy: Validation of a 3D Skeletal Muscle Organoid Platform

**DOI:** 10.3390/biomedicines13051109

**Published:** 2025-05-03

**Authors:** Raffaella Quarta, Enrica Cristiano, Mitchell K. L. Han, Brigida Boccanegra, Manuel Marinelli, Nikolas Gaio, Jessica Ohana, Vincent Mouly, Ornella Cappellari, Annamaria De Luca

**Affiliations:** 1 Department of Pharmacy Drug Science, University of Bari Aldo Moro, 70125 Bari, Italy; raffaella.quarta@uniba.it (R.Q.); brigida.boccanegra@uniba.it (B.B.); annamaria.deluca@uniba.it (A.D.L.); 2 BIOND Solutions B.V., 2629 JD Delft, The Netherlands; 3 Institut de Myologie, Centre de Recherche en Myologie, Sorbonne Université, 75013 Paris, France

**Keywords:** Duchenne muscular dystrophy, 3D skeletal muscle organoid, tissue engineering, immortalized human myoblast, disease modeling

## Abstract

**Background:** Three-dimensional skeletal muscle organoids (3D SkMO) are becoming of increasing interest for preclinical studies in Duchenne muscular dystrophy (DMD), provided that the used platform demonstrates the possibility to form functional and reproducible 3D SkMOs, to investigate on potential patient-related phenotypic differences. **Methods**: In this study, we employed fibrin-based 3D skeletal muscle organoids derived from immortalized myogenic precursors of DMD patients carrying either a stop codon mutation in exon 59 or a 48–50 deletion. We compared dystrophic lines with a healthy wild-type control (HWT) by assessing microtissue formation ability, contractile function at multiple timepoints along with intracellular calcium dynamics via calcium imaging, as well as expression of myogenic markers. **Results**: We found patient-specific structural and functional differences in the early stages of 3D SkMO development. Contractile force, measured as both single twitch and tetanic responses, was significantly lower in dystrophic 3D SkMOs compared to HWT, with the most pronounced differences observed at day 7 of differentiation. However, these disparities diminished over time under similar culturing conditions and in the absence of continuous nerve-like stimulation, suggesting that the primary deficit lies in delayed myogenic maturation, as also supported by gene expression analysis. **Conclusions**: Our results underline that, despite the initial maturation delay, DMD muscle precursors retain the capacity to form functional 3D SkMOs once this intrinsic lag is overcome. This suggests a critical role of dystrophin in early myogenic development, while contraction-induced stress and/or an inflammatory microenvironment are essential to fully recapitulate dystrophic phenotypes in 3D SkMOs.

## 1. Introduction

Duchenne muscular dystrophy (DMD) is a muscle-wasting, X-linked recessive disorder affecting 1 in 5000 live male births [1,2]. The disease leads to severe progressive loss of muscle contractile ability, cardiomyopathy, respiratory failure, and premature death [3,4]. DMD is caused by heterogeneous mutations in the gene encoding for the dystrophin protein (DMD), one of the largest proteins in the human body, and its loss leads to escalating muscle tissue waste and consequent loss of function [5]. In striated muscle, dystrophin links the cytoskeleton to the extracellular matrix, reinforcing the sarcolemma during the stress of contraction and exerting a key role in intracellular signaling. In fact, dystrophin interacts directly or indirectly with several partners, including sarcolemma and extracellular proteins, cytoskeletal components (actin microfilaments, microtubules, and other related structural/scaffolding proteins), and other cytolinkers. Dystrophin and its direct binding partners form the dystrophin associated protein complex (DAPC) [4]. The lack of dystrophin leads to the disassembly of this complex, causing the loss of interaction between F-actin and the extracellular matrix and disturbance in mechano-transduction signaling, triggering a complex cascade of pathogenic events with progressive muscle wasting. Alterations of calcium homeostasis, impaired metabolism, and over-production of reactive oxygen species along with chronic inflammation and fibrosis are typical hallmarks of dystrophic muscle and contribute to progressively impair muscle function [6,7]. Many innovative approaches have been and still are under investigation to advance DMD therapy, focusing either on restoring the dystrophin protein production or on abating the complex pathophysiological cascade triggered by its absence [8,9]. Currently, gene therapy and antisense oligonucleotides for mutation-specific exon skipping are at advanced stages of development, while some small molecules targeting downstream events, such as dissociative steroid vamorolone [10] and histone deacetylase inhibitor givinostat [11], have been recently approved for clinical use. However, important limitations in effectiveness need to be overcome, mostly in relation to multiple mutations, the complexity of the disease cascade, the presence of disease modifiers, and patient genetic background. Accordingly, preclinical drug studies in DMD are still compelling. In this context, the *mdx* mouse has been the most widely used murine model of DMD and has contributed over the last forty years to greatly advancing the understanding of the role of dystrophin and in developing some promising approaches [12,13,14,15,16] However, the *mdx* mouse suffers from the common limitations of animal-based translational research, such as high costs, long timelines, ethical concerns related to the large number of animals required, and low throughput [17]. The use of 2D cultures from human-derived muscle precursors, especially for a complex disease like DMD, are scarcely predictive of the activity and structure of differentiated skeletal muscle and therefore also in the assessment of drug efficacy on clinically relevant readouts [18,19].

Three-dimensional skeletal muscle organoids (3D SkMOs) obtained from patient-derived immortalized cell lines represent an attractive strategy in DMD pre-clinical research, as they have the potential to recapitulate, in vitro, the physiological complexity of tissue differentiation and function, as well as relevant disease-related pathophysiological mechanisms. This can accelerate the identification of new or repurposed drug candidates with potentially beneficial therapeutic outcomes while considering the 3R principles (Refine, Replace, and Reduce) for the ethical reduction of animal use [20,21]. In the last decade, important advancements have been made in the field of engineering skeletal muscle tissues using various devices and multiple cell types. For instance, miniaturized 3D myotube cultures on chips, derived from immortalized human cell lines, were used to model LMNA-related congenital muscular dystrophy (L-CMD), allowing assessment of the impact of diseases on muscle structure and force production [22]. Similarly, promising artificial skeletal muscle tissues were fabricated from human pluripotent stem cells (iPSCs) from patients with Duchenne, limb-girdle, and congenital muscular dystrophies [23]. The possibility of having artificial muscle organoids generated from different DMD patients paves the way to continue phenotype-directed studies in relation to different mutations and genetic backgrounds. Few studies are available in this regard, and the high heterogeneity in the approaches used by different laboratories—regarding scaffold/support, cell sources, methods for contraction determination and/or the use of more advanced dynamic microfluidic organ-on-chip technology—hinders easy comparison between data or standardization of procedures. In particular, the 3D muscle bundles obtained by immortalized myoblast lines established from healthy and DMD donors show a minor trend towards contractile weakness unless a damaging stressor is used, with minimal, if any, phenotype-related differences when investigated [24,25]. In contrast, in the paper by Nesmith et al., it was found that a dystrophic 3D tongue organoid developed significant weakness compared to healthy tissue but no phenotype-specific comparison could be made [26]. On the other hand, silencing dystrophin with siRNA leads to complete loss of contraction ability in 3D organoids [27], a condition that contradicts the notion that dystrophin is not, per se, essential for contractile machinery. In this scarce and new scenario, it is fundamental to use different platforms in order to assess the possibility of comparable results for the main disease-related alterations in the 3D environment, meanwhile progressing towards the standardization of human-derived 3D skeletal muscle organoid platforms for in vitro studies.

To this aim, we performed an exploratory assessment using two human-derived immortalized muscle cell lines carrying different mutations in the DMD gene, DMD1, from a 11-year-old patient carrying a stop codon mutation on exon 59, and DMD2, from a 14-year-old patient carrying a 48–50 deletion, and a control line (HWT) from a 16-year-old healthy donor, to highlight possible functional differences in the development of 3D SkMOs related to different genetic backgrounds. Our results support the view that intrinsic differences mainly impact the velocity of 3D SkMO differentiation, although dystrophic muscle precursors are able to efficiently produce bundles with comparable contractile performance. Then, data confirm that a proper disease-like environment and stimulation must be recreated for mimicking the long-term patient-like contraction defects and wasting profile and, eventually, phenotypic alterations.

## 2. Materials and Methods

### 2.1. Human Immortalized Cell Lines Maintenance

To develop 3D SkMOs, we had access to human-derived immortalized cell lines, granted from the biobank MyoLine, previously immortalized by Vincent Mouly lab: HWT (AB1190), DMD1 (stop exon 59; AB1023), and DMD2 (deletion 48–50; AB1098). Due to the exploratory nature of the present study, which is mainly to verify the quality and main functional profile of 3D SkMOs, three conditions (1 WT and two different DMD mutations) were considered sufficient. Cells were cultured in flasks and maintained in a proliferative state (myoblast) at 37 °C using a proliferation medium comprising basal medium (Skeletal muscle cell growth medium; PromoCell Heidelberg, Germany C-23260), 15% FBS Fetal Bovine Serum (Gibco; São Paulo, Brazil, 10270106), 1% gentamicin (Gibco—15750037), 1% GlutaMax (Gibco 35050038), and 5% growth medium supplement mix (PromoCell C-39365). Differentiation was initiated by using a differentiation basal medium (Skeletal muscle differentiation medium; PromoCell C-23061) with the addition of 2% differentiation medium Supplemental mix (PromoCell C-23061) and 2% FBS (Gibco).

### 2.2. 3D SkMO Formation

Three-dimensional SkMOs were formed using the commercially available MUSbit™ system (BIOND Solutions B.V., Delft, The Netherlands), a chip designed with an oval-shaped open well containing two pillars that allow muscle fiber alignment (Figure S1). A modified design of the chip (MUSbit-1C^TM^) consists of a silicon-based chip attached to a PDMS-based membrane, creating a well compartment and a microfluidic channel separated by a thin, porous membrane. A similar chip with the exact same microfluidic channel design and fabrication method has previously been described [28,29,30]. For initial testing, chips without a microfluidic channel (MUSbit) were fabricated and mounted in a custom multi-well plate format (Figure S2). Chips were previously plasma treated to enhance hydrophilicity and coated with 5% Pluronic^®^ F-127 (Sigma-Aldrich, Saint Louis, MO 63103, USA, P2443) solution in PBS (or 5% BSA in PBS). This step is useful to keep the chips hydrophilic but should also prevent cells from sticking to the PDMS. Plasma-treated chips were first sterilized using 70% IPA/Ethanol and incubated with coating solution overnight. Cells embedded in hydrogel mixture were seeded into the chip well at a final concentration of 1.50 × 10^7^ cells/mL. For each condition (HWT, DMD1, and DMD2), cells derived from different flasks were used in order to minimize any potential experimental bias. Fibrin/Geltrex hydrogels are formed by mixing fibrinogen (Sigma-Aldrich F3879-1G) and Geltrex™ (Gibco™A1413202) with thrombin (Sigma-Aldrich T4648-1KU). Two mixtures were prepared and kept on ice. The composition of the mixture is shown in Table 1. Aprotinin (Sigma-Aldrich A1153-10MG) was also added into the mix to prevent fibrin degradation. Gelling solution was added to the cell solution and mixed thoroughly; then, 2 μL of the solution was individually pipetted within the chip well. The cell/hydrogel mixture was polymerized for 20 min at 37 °C, followed by incubation in growth media. In parallel, the same number of seeded cells were cultured in 2D to assess the amount, if any, of growth over the 24 h. None of the three cell lines exhibited substantial growth nor death during this period. After 24 h, cells were incubated in differentiation medium containing 2.0 mg/mL 6-aminocaproic acid (ACA, Sigma-Aldrich A2504), an antifibrinolytic agent. Three-dimensional SkMOs were maintained in culture until each timepoint, refreshing every 48 h with differentiation medium supplemented with 2.0 mg/mL 6-aminocaproic acid. Furthermore, to evaluate the reproducibility of the system, we screened the entire population of 3D SkMOs obtained, according to several morphological parameters carefully described in Table S1, to identify the percentage that met the acceptance criteria. Three-dimensional SkMOs considered unacceptable were, therefore, excluded from the analysis. The 3D SkMOs were then used either for contraction recordings, calcium transient analysis, or molecular biology. Considering that microtissues were formed from the same number of cells and that their lengths were determined by the distance between the pillars, their primary difference could be in the diameter that was measured at the midpoint for each tissue using Fiji ImageJ. These diameters were also used to normalize the contraction force of 3D SkMOs.

### 2.3. Contraction Assay on the Multi-Well Plate Format

Three-dimensional SkMOs were electrically stimulated to elicit contraction at three different timepoints: day 7, 9, and 13 of differentiation. These timepoints for stimulation and functional assessments were arbitrarily selected on the basis of the following consideration: 7 days, as a minimum lag time to allow bundle formation in quiescent state; 9 days, to assess the speed of developmental rate; 13 days, as a later timepoint after a short-term quiescent state. A special lid containing 2 platinum electrodes was developed for muscle stimulation. These electrodes were connected to a custom-made electrical stimulation device containing a pulse generator controlled by in-house developed proprietary software to generate biphasic electrical pulses (provided by BI/OND Solutions B.V.) (Figure S2). Briefly, 3D SkMOs were stimulated with a single 10 ms pulse at 5 V (twitch) and a train of 10 ms pulses at 5 V and 30 Hz frequency for 3 s (tetanus). Fatigability was also evaluated by exposing 3D SkMOs to a train of short tetanus stimulations (1200 ms pulse at 30 Hz every 5 s) for 1 min and then calculating the percentage of force between all pulses compared to the first one. Muscle contractions were recorded at 100 ms intervals using a camera (Pixelink M2-CYL-PixeLINK uScope Standard) coupled to an inverted microscope in brightfield mode (Euromex Oxion Inverso-OX.2003-PL). Due to the design of our device, contraction amplitude was measured using an automated, open-source software tool (MUSCLEMOTION_v1_.ijm) that allows rapid and easy measurement of movement detected from high-speed movies through pixel displacement [31,32,33]. Contraction forces were normalized upon 3DSkMOs’ diameter, assuming that this latter would, with good approximation, recapitulate the distribution of myofibers in the central part [34].

### 2.4. Imaging of Calcium Transients

To measure calcium transients, experiments were performed in a live imaging chamber at 37 °C and 5% CO_2_ to maintain cells in physiological conditions during recording. For imaging, FluoroBrite DMEM (Gibco—A1896702) was supplemented with 1% GlutaMAX (100× Gibco 35050-038), 1% Penicillin-Streptomycin (5000 U/mL Gibco 15070-063), and 1% Sodium Pyruvate (100× Gibco 11360070). Samples were incubated with 200 µL Fluo-4 loading solution (Fluo-4 AM Thermo Fisher F14201) for 1 h. Before starting imaging, samples were washed and then incubated with imaging medium for an additional 15 min at 37 °C. Fluorescent images were acquired using a Nikon Eclipse TI2-E inverted fluorescence widefield microscope coupled to a D-LEDI Fluorescence LED illumination system and a Digital Sight 10 camera. Images were captured with a Nikon CFI Plan Fluor 4× objective (NA 0.13) using the GFP channel (475 nm at 12% power excitation using a GFP-4050B filtercube). The camera captured images with 3 × 3 binning, 50 ms exposure time, and 9.3× gain at 20 fps for a duration of 6 s. Calcium transients were obtained through the application of electrical stimulation pulses at frequency ranges of 1 Hz, 2 Hz, 5 Hz, 10 Hz, and 30 Hz. Analysis was performed using FiJi (ImageJ) software Java 1.8.0_322, and the fluorescence signal was calculated by using the following formula:

$$\frac{\Delta F_{t}}{F_{0}}=\frac{F_{t}-F_{0}}{F_{0}}.$$

*F*_0_ is a measure of the baseline fluorescence at the resting state, and Δ*F*_t_ is the moment-by-moment deviation from that baseline.

### 2.5. Immunofluorescence Staining

Three-dimensional SkMOs were immunostained for myosin II heavy chain and sarcomeric α-actinin. Following fixation in 4% paraformaldehyde in PBS for 15 min at room temperature, samples were washed 3 times with PBS and blocked in Blocking Buffer (2% BSA + 0.3% Triton X-100) for 30 min. Primary antibodies were diluted in Blocking Buffer (w/Triton) and incubated overnight at 4 °C. A secondary antibody solution with DAPI and Phalloidin-488 was added the day after and incubated for 2 h. Samples were mounted in Mounting Medium (Ibidi Mounting Medium) before imaging. Primary antibodies: SAA (sarcomeric α-actinin—Sigma A7811) 1:1000; MF20 (myosin II heavy chain—DSHB) 1:50. Secondary antibody solution: Alexa 647 Goat anti-Mouse (1:200—Thermo Fisher A21235); Phalloidin-Atto 488 (1:200—Sigma 49409); DAPI (0.5 µg/mL—Sigma D9542). Images were acquired using a Nikon TI2-E confocal microscope setup, coupled with a Crest X-light V3 Spinning disk unit (50 μm pinhole size and 250 μm pinhole spacing), a Photometrics Kinetix sCMOS camera, and a CELESTA light engine (Lumencor, Beaverton, OR, USA). Images were acquired using a Nikon 20× Plan Fluor multi-immersion objective (0.75 NA, set at 0.33 Water Immersion) with 3 µm steps in the z-direction. The following settings were used to acquire the images: DAPI (405 nm 40% laser power, 500 ms exposure), Phalloidin (488 nm 20% laser power, 400 ms exposure), MF20/SAA (640 nm 21.3% laser power, 400 ms exposure). Data were processed and analyzed using Fiji ImageJ Java 1.8.0_322.

### 2.6. RNA Isolation, cDNA Synthesis, and Real Time-PCR

To obtain a sufficient amount of material for adequate RNA isolation, it was necessary to pool four 3DSkMOs together per cell type; each biological replicate was therefore representative of four different samples. Total RNA was isolated using a Total RNA Purification Plus Micro Kit (Norgen Biotek, Ontario, CanadaCorp. NR48500) following the manufacturer’s instructions from 3D SkMOs differentiated to day 13. RNA purity was evaluated via nanodrop spectroscopy to confirm 260/280 ratios were >2.0 and 260/230 ratios were >1.8. cDNA synthesis was performed using the iScript™ gDNA Clear cDNA Synthesis Kit (Bio-Rad) as follows: RNA samples were first DNase treated using iScript DNase and iScript DNase Buffer, according to the manufacturer’s protocols. Following DNase heat inactivation, samples were reverse transcribed using oligodT and random priming. Quantitative Real Time PCR was performed via the CFX384 Connect Real-Time PCR system (Bio-Rad, Hercules, CA, USA) by using Prime PCR assays SYBR^®^ green (cod. 10025636). Each reaction was performed in technical triplicate in Hard-shell^®^ 96-well PCR plates. All gene primers were purchased from Bio-Rad (Table 2). The mRNA expression of genes was normalized to the mean of the two housekeeping genes (RPS18 and UBC), with sample normalizer selection and quantification made using Bio-Rad CFX Maestro software(version 5.3.022. 1030).

### 2.7. Microfluidic Setup

The MUSbit-1C^TM^ chip (BIOND Solutions B.V.) features a porous channel that allows controllable circulation of liquid through the microfluidic channel to perfuse the tissue (Figure S1). The chip was mounted on the comPLATE^TM^, an interface that allows connection to a perfusion system. The interface was connected to an internally developed pneumatic device that introduces fluidic unidirectional flow using a commercially available micropump (Bartels mp6—Bartels Mikrotechnik GmbH, Germany) (Figure S3). We chose a speed of 100 μL/min, higher than what have been used in other studies [35,36] but allowing a faster solution replacement; this speed was well tolerated in our system. The lid of the device contained 2 platinum electrodes at a distance of 1.2 mm, which were connected to an internally developed pulse generator (BIOND Solutions BV) (Figure S4). This allowed simultaneous perfusion and electrical stimulation of the 3D SkMOs. Initial characterization of the flow into the MUSbit-1C^TM^ chip was performed by measuring FITC-Dextran (Sigma FD40S) during flow using a Nikon Eclipse TI2-E inverted fluorescence widefield microscope coupled to a D-LEDI Fluorescence LED illumination system and a Digital Sight 10 camera. Images were captured with a Nikon CFI Plan Fluor 4× objective (NA 0.13) using the GFP channel (475 nm at 12% power excitation using a GFP-4050B filtercube, 3 × 3 binning, 400 ms exposure time, 2.2 gain). The average fluorescence intensity at any given timepoint was determined by at least 3 ROIs per field of view. Three-dimensional SkMO contraction was monitored with the same microscope setup using a Nikon CFI Plan Fluor 4× objective (NA 0.13) using the brightfield channel (12.7% DIA, 3 × 3 binning, 100 ms exposure, 7.6× gain) at 10 fps. The microscope stage was enclosed with an incubation chamber and connected to a heating/CO_2_ system (Okolab), keeping samples, as well as inlet and outlet reservoirs at 37 °C and a 5% CO_2_ atmosphere during the entire duration of the experiment. For contraction inhibition, the inlet reservoir contained a 10 mM solution of 2,3-Butanedione monoxime (BDM), a non-competitive inhibitor of skeletal muscle myosin-II. The solution was also spiked with 0.125 mg/mL FITC-Dextran as a proxy to monitor the presence of inhibitors during the experiment. Data were analyzed using Fiji ImageJ software (Java 1.8.0_322)

### 2.8. Statistical Analysis

All data were expressed as mean ± standard error of the mean (SEM). The normality of the data was assessed via the Shapiro–Wilk normality test (alpha = 0.05). For data with normal distributions, multiple statistical comparisons between groups were performed using one-way ANOVA followed by Dunnett’s post hoc correction. For non-parametric analysis, Brown–Forsythe ANOVA analysis with Dunnett’s multiple comparisons was performed.

## 3. Results

### 3.1. Monitoring 3D SkMO Formation and Assessment of Reproducibility

**Figure 1:**
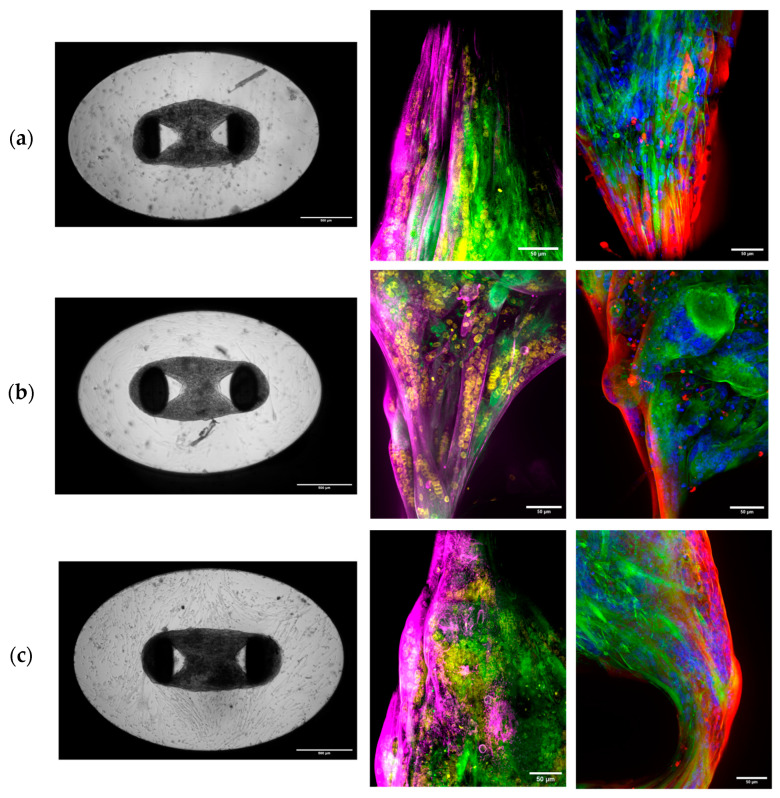
Representative images of 3D SkMOs on day 13 of differentiation. On the left: (**a**) HWT, (**b**) DMD2, and (**c**) DMD1. Scale bar 500 μm. Confocal immunofluorescence imaging of tissues’ sections at day 9 of differentiation showing sarcomeric α-actinin (magenta), F-actin (green), and nuclei (yellow) on the left panel and myosin II heavy chain (red), F-actin (green), and nuclei (blue) on the right. Apparent aggregates, due to unspecific staining, are noticeable in some images. Scale bar 50 μm. Immunofluorescence image of a full-length 3D SkMO is shown in Figure S5.

**Figure 2:**
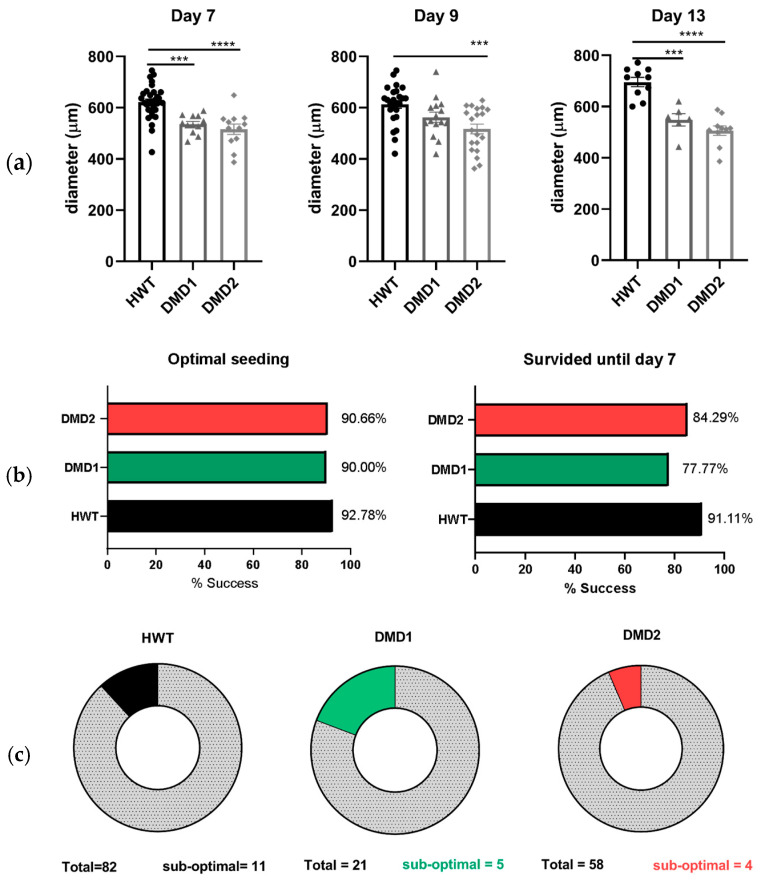
(**a**) Bar graphs presenting diameter values for each 3D SkMO at every timepoint. All data are presented as mean ± standard error of the mean (SEM). Multiple statistical comparisons between groups were performed by ordinary one-way ANOVA analysis with Dunnett’s multiple comparisons post hoc correction. Day 7: HWT vs. DMD1 *** *p* = 0.0003, HWT vs. DMD2 **** *p* < 0.0001. Day 9: HWT vs. DMD2 *** *p* = 0.0003. Day 13: HWT vs. DMD1 *** *p* = 0.0001, HWT vs. DMD2 **** *p* < 0.0001. (**b**) Bar graphs showing percentage of seeding success (left) and percentage of survival until 7 (right). (**c**) Pie charts showing the sub-optimal population for each cell line.

Three-dimensional SkMOs were formed using immortalized myoblasts from a healthy donor (HWT, used as control), a patient-derived dystrophic line with stop codon mutation on exon 59 (DMD1), and a patient-derived dystrophic line with a 48–50 deletion (DMD2). For the seeding, the same number of cells, equal to 30.000 per gel, was used in each condition. Two hours after seeding, the hydrogel had already started to compact, and on day 2, 3D SkMOs were completely formed around the two pillars. At day 9 of differentiation, the 3D SkMOs had formed multinucleated myotubes; positive staining of sarcomeric α-actinin (SAA) and myosin II heavy chain, both early markers of muscle fiber differentiation, was also appreciable, especially around the pillars. (Figure 1). The formation of myotubes could be observed via the confocal imaging of 3D SkMOs, regardless of the donor cell type (Figure 1). As a readout for 3D SkMO compaction and tissue self-organization, we examined 3D SkMO shape during compaction by measuring the diameter, perpendicular to tissues’ length, of each tissue from brightfield images (4× magnification). Interestingly, we found a statistically significant difference between the diameters of control and dystrophic 3D SkMOs, suggesting a possible alteration in tissue organization and/or myocyte fusion efficiency between cell lines (Figure 2a). As anticipated in the methods section, this difference was not related to a different number of myoblasts forming the 3D SkMOs, since the growing rate is minimal and comparable between the different conditions after 24 h. Furthermore, we evaluated the reproducibility of the system by quantifying the rate of success of 3D SkMO formation and, among those, the survival after 7 days of seeding in a quiescent state (minimum timepoint request for each experiment) (Figure 2b). To do so, we screened the entire population obtained according to several parameters (Table S1) to identify the percentage of them matching the acceptance criteria. Three-dimensional SkMOs considered sub-optimal, which represented only 13% (HWT), 23% (DMD1), and 6% (DMD2) of the entire populations, were considered acceptable and hence included in the analysis, while 3D SkMOs considered unacceptable were excluded from the analysis (Figure 2c).

### 3.2. Functional Analysis Highlights Distinct Contraction Properties Among Cell Lines

**Figure 3:**
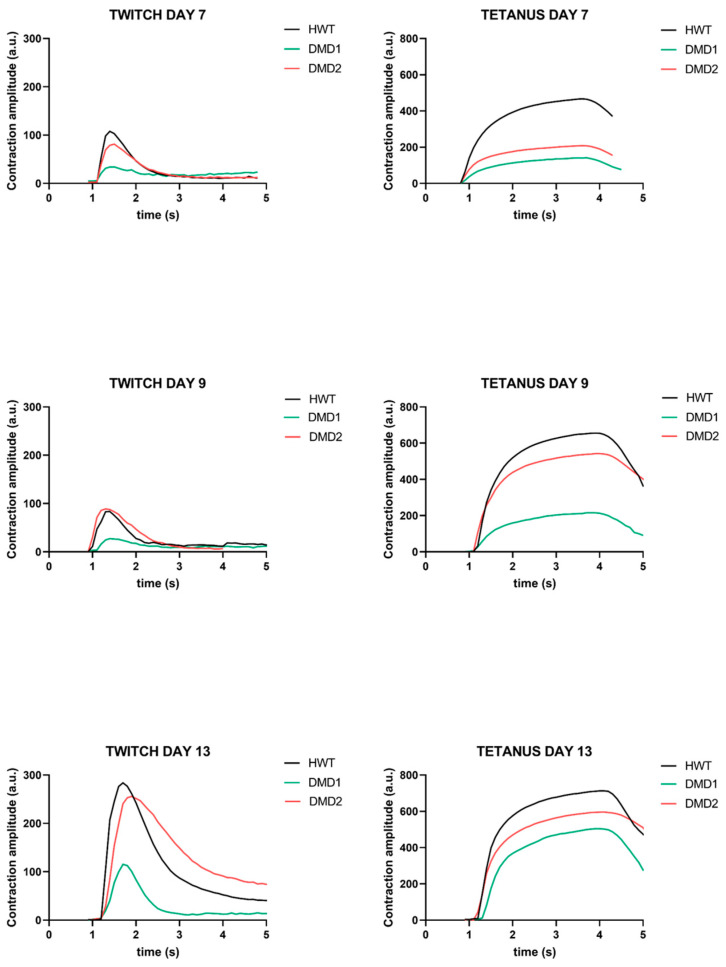
Representative contraction curves in response to EPS showing twitch-like contractions (on the **left**) and tetanic contractions (on the **right**) overlapping for each line.

**Figure 4:**
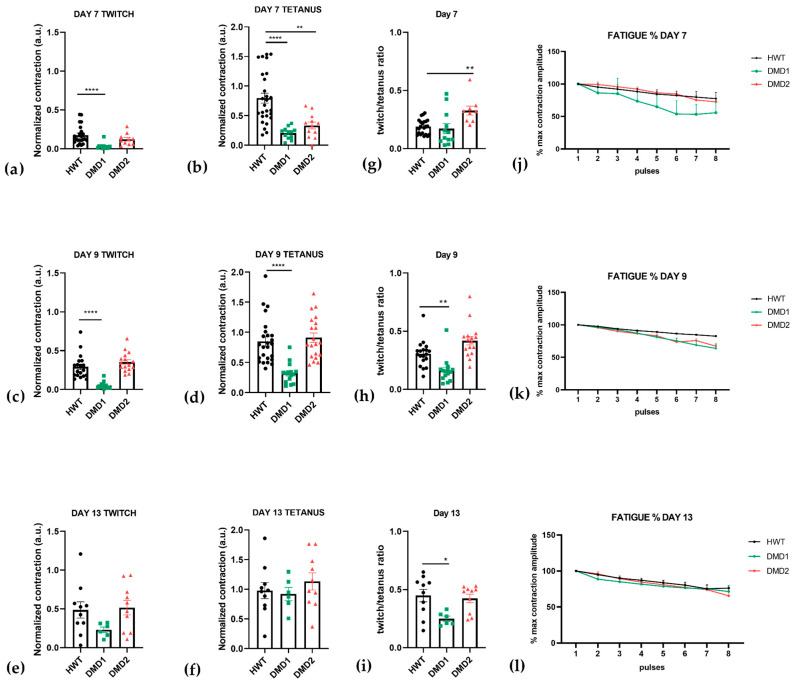
Maximum contraction values for twitch (single 10 ms pulse at 5 V) and tetanic responses (5 V, 10 ms pulses at 30 Hz) at three differentiation timepoints. Each 3D SkMO’s contraction amplitude was normalized using their diameter. (**a**,**b**) Day 7: twitch (HWT *n* = 22, DMD1 *n* = 12, DMD2 *n* = 10), tetanus (HWT *n* = 27, DMD1 *n* = 12, DMD2 *n* = 12). (**c**,**d**) Day 9: twitch (HWT *n* = 25, DMD1 *n* = 15, DMD2 *n* = 17), tetanus (HWT *n* = 25, DMD1 *n* = 15, DMD2 *n* = 20). (**e**,**f**) Day 13: twitch (HWT *n* = 10, DMD1 *n* = 6, DMD2 *n* = 10), tetanus (HWT *n* = 10, DMD1 *n* = 6, DMD2 *n* = 10). (**g**–**i**) Twitch/Tetanus ratio for each timepoint. (**j**–**l**). Fatigue was measured in a bundle subset as the drop of force between each pulse compared to the first one in percentage of the maximum force generated at each pulse. Day 7: HWT *n* = 3, DMD1 *n* = 4, DMD2 *n* = 3. Day 9: HWT *n* = 6, DMD1 *n* = 5, DMD2 *n* = 4. Day 13: HWT *n* = 4, DMD1 *n* = 3, DMD2 *n* = 4. All data are expressed as mean ± SEM. Multiple statistical comparisons between groups were performed by one-way Brown–Forsythe ANOVA analysis with Dunnett’s multiple comparisons test post hoc correction: * *p* = 0.0191, ** *p* < 0.01, **** *p* < 0.0001.

**Figure 5:**
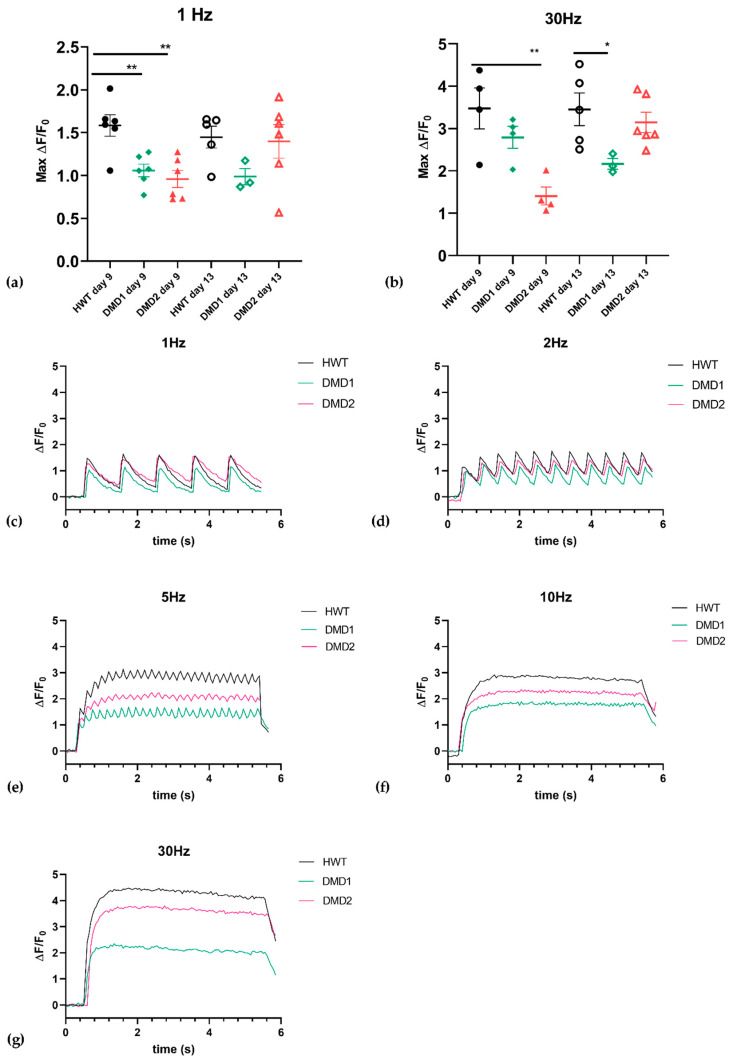
Calcium transient values expressed as maximum values of normalized fluorescent signal elicit by (**a**) 1 Hz and (**b**) 30 Hz EPS. Data are presented as mean ± (SEM): 1 Hz day 9: HWT (*n* = 6), DMD1 (*n* = 6), DMD2 (*n* = 6); 30 Hz day 9: HWT (*n* = 4), DMD1 (*n* = 4), DMD2 (*n* = 4); 1 Hz day 13: HWT (*n* = 5), DMD1 (*n* = 3), DMD2 (*n* = 6); 30 Hz day 13: HWT (*n* = 5), DMD1 (*n* = 3), DMD2 (*n* = 6). Significant differences were assessed by one-way ANOVA with Dunnet’s multiple comparison correction. (**a**) One hertz at day 9: HWT vs. DMD2 ** *p* = 0.0011, HWT vs. DMD1 ** *p* = 0.0045. (**b**) Thirty hertz at day 9: HWT vs. DMD2 ** *p* = 0.0036. (**b**) Thirty hertz at day 13: HWT vs. DMD1 * *p* = 0.038. (**c**–**g**) Calcium transient responses to EPS through the application of a frequency range of 1 Hz, 2 Hz, 5 Hz, 10 Hz, and 30 Hz at day 13.

To evaluate muscle functionality, 3D SkMOs were subjected to electrical pulse stimulation (EPS) to induce twitch-like and tetanus contractions at days 7, 9 and 13 of differentiation (Figure 3). Videos of contraction are shown in the Supplementary Materials (Videos S1a–f). Differences in the contractile behavior between control and DMD samples were measured by calculating maximum twitch, tetanic forces, twitch-to-tetanus ratio, and fatigability. Subsequently, each diameter was used to normalize contraction maximum values. All 3D SkMOs displayed increasing responses to EPS in a timepoint-related manner, showing that contraction force increases as differentiation progresses. On day 7 (Figure 4a,b), HWT showed higher twitch and tetanic (with 30 Hz stimulus) contraction forces than dystrophic lines, which displayed lower responses. On day 9 (Figure 4c,d), however, DMD2 3D SkMOs performed similarly to healthy ones, showing no relevant phenotype-related difference at this timepoint, while DMD1’s values were still significantly lower than HWT. On day 13 (Figure 4e,f), no significant differences in EPS were observed between the cell lines. The twitch-to-tetanus ratio of each timepoint displayed lower values for DMD1 compared to HWT, whereas DMD2 showed similar and even statistically higher values on days 7 and 9 compared to HWT. Furthermore, to have a first assessment of fatigability, we exposed 3D SkMOs to short trains of tetanic stimulation (10 ms pulses at 30 Hz) with 5 s intervals between each other for one minute. All 3DSkMOs displayed a certain drop in force between the first and last pulses at every point, which was more evident in DMD1 at day 7 compared to HWT and DMD2 (Figure 4j–l). However, this difference was less appreciable on day 9. Moreover, we evaluated intracellular calcium transient responses to EPS through the application of a frequency range of 1 Hz, 2 Hz, 5 Hz, 10 Hz, and 30 Hz (Video S2). We observed calcium transients mimicking twitch, unfused tetanus, and complete tetanic responses, in which it can be appreciated how calcium flux curves are consistently higher in HWT than in both dystrophic 3D SkMOs at day 13 (Figure 5c–g). On day 9, DMD2 and DMD1 calcium transients were significantly lower than that for HWT. However, by day 13, no significant difference between cell lines was observed (except for DMD1 values at 30 Hz), showing that, especially for DMD2, contraction-matched transient values returned to as high as those for HWT (Figure 5a,b). The calcium transients for HWT and DMD1 at the various timepoints are in good agreement with the twitch/tetanic ratios, while the discrepancy in DMD2 presently remains less clear. Altogether, the evaluation of contractile responses and calcium imaging transients support the view that, over time, the 3D SKMOs progressively acquired the clues of the excitation–contraction coupling mechanism. Also, while all 3D SkMOs improved their contraction strength as they matured, healthy 3D SkMOs outperformed dystrophic ones at early timepoints.

### 3.3. Gene Expression of Myogenesis Markers

**Figure 6:**
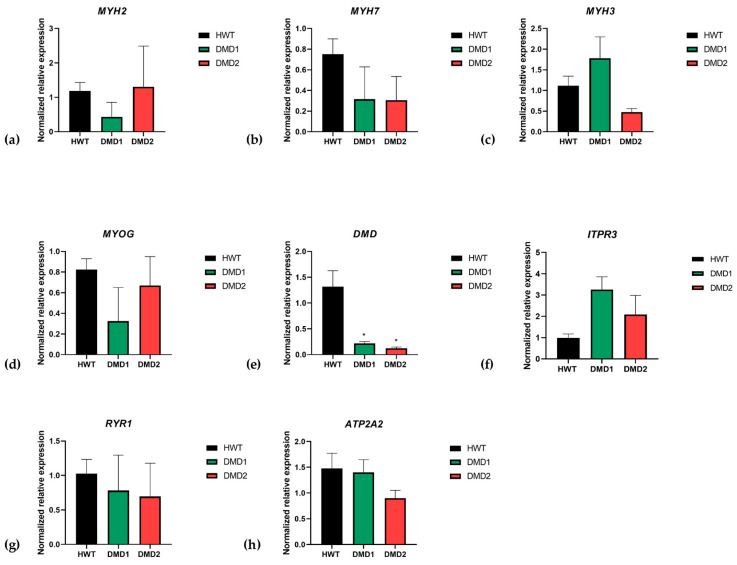
RT-PCR analysis in muscle microtissues of (**a**) *MYH2* (HWT *n* = 3, DMD1 *n* = 3, DMD2 *n* = 2), (**b**) *MYH7* (HWT *n* = 3, DMD1 *n* = 2, DMD2 *n* = 2), (**c**) *MYH3* (HWT *n* = 3, DMD1 *n* = 3, DMD2 *n* = 2), (**d**) *MYOG* (HWT *n* = 3, DMD1 *n* = 2, DMD2 *n* = 2), (**e**) *DMD* (HWT *n* = 3, DMD1 *n* = 3, DMD2 *n* = 2) HWT vs. DMD 1 (* *p* = 0.0183) and HWT vs. DMD2 (* *p* = 0.0207), (**f**) ITPR3 (HWT *n* = 3, DMD1 *n* = 2, DMD2 *n* = 2), (**g**) *RYR1* (HWT *n* = 3, DMD1 *n* = 3, DMD2 *n* = 2), and (**h**) ATP2A2 (HWT *n* = 3, DMD1 *n* = 2, DMD2 *n* = 2). Data are expressed as mean ± SEM. Significant differences were assessed by ANOVA with Dunnet’s multiple comparison correction.

Finally, to evaluate the 3DSkMOs’ maturation states on day 13 of differentiation, we analyzed the gene expression of myogenic factors and key muscle contractility proteins through RT-PCR. The *MYH2* (2a fast-twitch fibers’ myosin) gene showed a different trend of expression between the two dystrophic lines, showing a lower expression in DMD1 than DMD2 and HWT (Figure 6a). *MYH7* (type I skeletal muscle fibers, also known as slow-twitch fibers’ myosin) showed a lower but not significantly different expression in both DMD1 and DMD2 samples compared to HWT (Figure 6b). Interestingly, as shown in Figure 6c, *MYH3* (embryonic form of myosin heavy chain) was more expressed in DMD1 compared to HWT, suggesting that DMD1 might exhibit a slower, delayed, or incomplete maturation process. Accordingly, *MYOG* (Figure 6d) was less expressed in DMD1 compared to HWT and DMD2, even though there was no statistical significance. As expected, the expression of the *DMD* transcript was significantly less expressed in both dystrophic lines than in the healthy control line (Figure 6e). Moreover, *RYR1* (Ryanodine Receptor 1), encoding the highly specialized calcium release channel from sarcoplasmic reticulum and essential for effective skeletal muscle excitation–contraction coupling, exhibited a trend of slightly lower expression in both dystrophic samples compared to HWT, although these changes did not reach significance (Figure 6g). A similar pattern was observed for SERCA2 (*ATP2A2*), the sarcoplasmic reticulum calcium ATPase pump, whose expression displayed no significant differences between samples (Figure 6h). Moreover, we assessed the expression of *ITPR3*, known to be expressed in muscle precursors and involved in differentiation via calcium-related signaling [37]. Interestingly, our data showed a clear (although not significant) overexpression of ITPR3 in dystrophic samples, especially DMD1, compared to HWT (Figure 6f), again suggesting a delay in maturation for dystrophic 3D SkMOs.

### 3.4. Microfluidic Technology Recreates a Dynamic Environment

**Figure 7:**
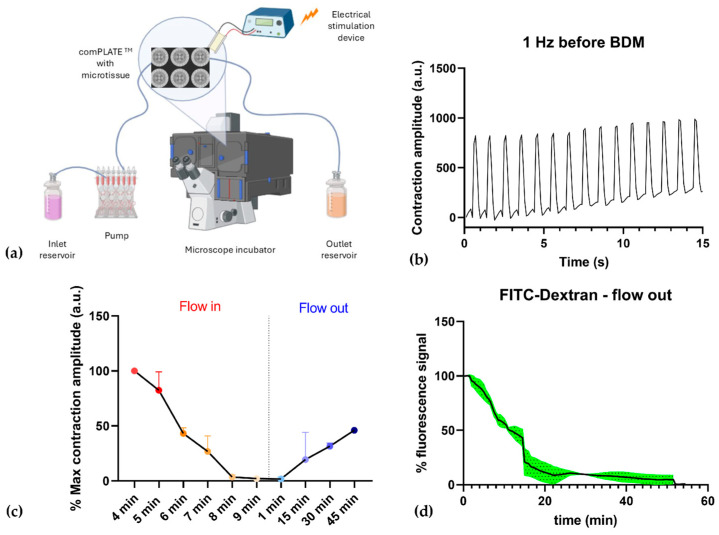
(**a**) Schematic representation of experimental setup: (**b**) One hertz contraction before introducing the inhibitor. (**c**) Contraction diminished over time after 4 min of BDM flowing in and contraction was restored over time during BDM wash out. (**d**) Fluorescence signal expressed as percentage decreasing during wash out. Data were collected from two independent experiments, and the values are presented as mean ± SEM (shown in green).

Once the procedure for contraction-competent 3D SkMOs was established, we were interested in validating the possible use of microfluidic technology. This holds potential applications in organ-on chip fields as it can effectively mimic perfusion and therefore be used for efficient drug administration. For this purpose, we assessed the possible pharmacological modulation of tissue contractility by applying 2,3-Butanedione monoxime solution (BDM), a myosin-II inhibitor, to HWT 3D SkMOs. These cells were chosen to avoid any bias in testing the microfluidic system due to intrinsic fragility of dystrophic myofibers. A schematic representation of the experimental setup is shown in Figure 7a, while authentic pictures of the set-up are in the Supplementary Materials (Figure S3). Data were collected from two independent experiments on day 8 of differentiation. We stimulated microtissues with 1 Hz electrical pulses at 5 V to produce twitch contraction responses (Figure 7b). Then, the BDM solution (10 mM) was introduced into the system with a flow rate of 100 µL/min; the solution also contained a fluorescent dye (FITC-Dextran Dye) to track liquid perfusion inside the chip well and to monitor drug wash out. Recording started 4 min after the flow began, thus this was set as time 0 (T0). Contraction was measured for 10 s every minute and, as shown in Figure 7c, it started diminishing progressively. At T = 9 min, the contraction amplitude dropped to 1.45% compared to the contraction at T0 and then reached a complete stop after 10 min. Subsequently, flow was paused to replace the inlet reservoir with only fresh medium. During the washout phase, the fluorescent intensity signal decreased until complete extinction after 45 min. In this phase, live contraction was acquired every 15 min and we detected that contraction slowly resumed concurrently with the decreasing fluorescence signal (Figure 7d). After 45 min, 46.08% of the initial contraction was restored, demonstrating that contraction was effectively modulated by the inhibitor introduced in the flow and not by the flux itself. The drug’s effects are expected to be reversible [38]; however, due to technical reasons, the experiments were stopped after 45 min, which was before full contraction was restored, suggesting that a longer time is required to observe complete recovery.

## 4. Discussion

While the use of 3D organoids is increasing in many fields, such as oncology and endocrine research, for both disease modeling and the testing of new therapeutics, the development of functional 3D skeletal muscle for neuromuscular disorders remains a significant challenge due to the intricate architecture and differentiation of muscle tissue and its direct relationship to function. Researchers have explored various strategies to overcome these obstacles, including the use of different biomaterials, different cell sourcing, scaffolding, and culturing techniques, and different stimulation and recording methods to replicate the structural and functional properties of native muscle [18,39]. While significant progress has been made in this field, the potential of 3D skeletal muscle organoids as a reliable disease modeling platform for predictive drug testing is still in its infancy. This is particularly true for Duchenne muscular dystrophy (DMD), the most common form of severe muscular dystrophy, which, over the last forty years, has attracted the main efforts of pre-clinical research, mainly through animal models [12,14,15,16]. The extensive studies in this field using classical models allowed researchers to gain insight into the complex role of dystrophin, including in relation to pathology presentation and progression, as well as to help develop new therapeutic approaches. While the extensive use of animal models is under debate for ethical reasons and the claims of low translatability, the milestones reached are pivotal when moving towards novel and poorly standardized platforms such as 3D in vitro models.

In the present work, we performed an independent explorative study to provide additional information on how, and if, 3D SkMOs obtained from immortalized patient-derived muscle precursors can reproduce structural and functional defects of interest for modeling DMD and its phenotypic variability in basal conditions, adding information to what has been observed in other similar platforms. Our main fundamental question also takes into account the long-standing debate about the intrinsic defect of satellite cells as the main determinant of regeneration failure in DMD [40], which opposes others’ data regarding dystrophic satellite cells being able to undergo an effective and efficient myogenic program. According to this last view, the main cause of the regeneration deficit would be satellite cell exhaustion due to the continuous cycle triggered by damage to the newly formed dystrophin-deficient myofibers, along with the hostile environment of inflammation and fibrosis, alongside niche disruption [41,42].

The results in the present study were obtained using healthy (HWT) and two dystrophic (stop codon mutation DMD1 and exons 48–50 deletion DMD2) cell lines that show a similar, although not fully overlapping, ability to obtain good quality tissues that are stable for up to 13 days in culture. A slight tendency to have smaller/thinner 3D SkMOs (at 13 days), along with a lower efficiency in 3D SkMO formation was observed. However, this also allowed us to confirm the reproducibility of our approach. While the difference in diameter was not due to a lag in the proliferation process, we hypothesized that variations in diameter were due to differences in myotube dimensions, as previously reported [24].

Importantly, we were able to carefully highlight key differences in muscle functionality between healthy (HWT) and dystrophic (DMD1 and DMD2) tissues, particularly in their ability to generate force and resist fatigue over time, even at early stages of formation. As expected, when tissues matured, all groups exhibited increased contraction strength. Both DMD1- and DMD2-derived 3D SkMOs displayed a delayed acquisition of contractile function, with a notable recovery over time that eventually reached the performance of healthy 3D SkMOs by day 9 for DMD2. DMD1-derived 3D SkMOs showed more pronounced deficiencies, characterized by weaker contractions and slower improvement in later stages, indicating a more delayed maturation. A similar trend was found for calcium transients. However, a clear recovery over culturing time was found, suggesting that the main defect is not related to the ability to form functional muscle tissues but rather to an intrinsic delay in entering the differentiation program. In line with this hypothesis, the expression of key myogenic genes was delayed, especially for DMD1. Notably, the altered ITPR3 expression is consistent with previous findings and further supports the role of this pathway in delaying myogenesis in dystrophic contexts [37].

Our results are in line with previous observations, corroborating that a possible fingerprint of dystrophic muscle precursors is the delayed efficiency to enter the myogenic program, a problem that is functionally overcome in the absence of stressors. For instance, muscle organoids were found by others to be contraction-competent after seven days in differentiation medium, with minimal, if any, phenotypic difference with respect to healthy controls or between different DMD mutations. In one study, dystrophic tissues were then used to recreate sarcolemmal damage in order to evaluate the therapeutic effect of candidate up-regulators of utrophin, a homologue of dystrophin [25]. A similar approach was described by Ebrahimi et al. in 2021, where they evaluated the efficiency of IgG- and integrin-activating therapies as a potential strategy to compensate for the role of dystrophin in stabilizing the sarcolemma against contraction-induced damage [24].

In our settings, fatigue tests further supported the hypothesis of the delayed maturation of dystrophic 3D SkMOs, in particular for DMD1, as there was a reduced ability to sustain contraction forces under repeated stimulation at early stages. However, defining and measuring fatigue is not straightforward due to the complex nature of the phenomenon and some limitations must be addressed before drawing a clear conclusion on real tolerance of dystrophic SkMOs; high frequency trains of stimulations and/or longer exposures may be needed.

Interestingly, in Nesmith et al.’s study, an in vitro model of tongue-on-chip from healthy and DMD donors was used to investigate cytoskeletal organization and myotube formation in DMD [26]. They hypothesized that DMD myoblasts might be less sensitive to ECM (extracellular matrix) cues that guide myotube alignment and questioned whether this slower alignment could result in delayed myofibrillogenesis and, consequently, weaker contractions. Concordant with our results, they found that these processes were indeed delayed in DMD tissues compared to healthy ones. A delay in the differentiation process has also been observed in our group in Cerchiara et al.’s study, which used 2D myoblast precursors from *mdx* mice when assessing the development of biophysical properties during differentiation [43]. Hence, the present study supports the interest in performing studies on 2D models to disclose intrinsic defects in the myogenic ability of patient-derived muscle precursors to better orient the preparation of predictive 3D SkMOs for personalized studies.

While some intrinsic differences in the myogenic program could be related to the underlying mutation via an unclear mechanism, we cannot rule out that differences in patient genetic background can also play a role. In this context, Iuliano et al. highlighted subtle differences in iPSC-derived 3D muscles from healthy subjects, likely in relation to genetic background, although key information, such as age, gender, ethnicity, etc., of the donors, was not disclosed [44]. Our early investigation into the suitability of the present platform to form functional 3D muscle organoids with only three main conditions, each one from single donor, need to be reinforced, including from a statistical point of view, with future studies using a larger number of both healthy and DMD-derived precursors (bearing the same or different mutations) and/or samples.

Also, we should disclose that the delay in differentiation and the rather short investigation time could have resulted in 3DSkMOs reaching a limited level of maturation and/or fiber alignments that could not be adequately solved by IF imaging. These findings emphasize the importance of using more complex and mature systems to reveal patient-related characteristics that might otherwise remain undetected and have an effect in clinical studies. The use of patient-derived cells, despite inter-individual variability, might better represent disease characteristics, reducing potential methodological biases. For instance, 3D tissues on a chip were also created by employing isogenic models obtained using a shRNA based knockdown strategy, inducing genetic defects for *DMD*, *CAPN3* (calpain-3), and *MSTN* (myostatin) [27]. The authors found that knockdown of *DMD* and *CAPN3* caused a reduction in contractile forces from day 3 followed by a complete absence of contractility by day 9. These findings are puzzling in view of the current knowledge on DMD pathophysiology. In fact, the absence of dystrophin does not per se impair muscle contraction, as patients acquire locomotor function that is maintained even up to 10–12 years of age. Then, the striking contraction failure observed in In ‘t Groen et al.’s study in 9-day differentiated tissues could be due to a combination of unclear factors that add to the impact of the induced genetic silencing.

In addition, as proof of concept of the potential of chips for dynamic applications, we tested our system using a microfluidic platform. While organ-on-chip (OOC) technologies are becoming increasingly attractive due to their ability to replicate in vivo organ function and drug pharmacokinetics, the development of muscle-on-chip microfluidic systems remains limited compared to other tissues. Previous research has demonstrated that skeletal muscle-on-a-chip platforms are valuable tools for studying tissue morphogenesis, maturation, and toxin- and drug-induced structural and functional changes. For example, in Fernández-Costa et al.’s study, a multi-organ-on-chip platform incorporating both muscle tissue and pancreatic islets was developed to investigate insulin secretion in response to muscle contraction [35]. Additionally, a muscle-on-chip system was established as a screening platform to study muscle injury in vitro. Researchers examined the dose-dependent effects of cardiotoxin on engineered muscle tissue architecture and its subsequent impact on passive tension, highlighting the applicability of such systems in studying tissue physiology and their potential use in drug discovery [45]. However, more detailed and focused experiments are needed to establish the main differences of dynamic vs. static cultures in our platform. Indeed, the standardization of these organ-on-chip systems may improve in vitro studies by recreating a more physiological environment and/or allow for the delivery of drugs of interest.

In the present study, we implemented a microfluidic approach to modulate tissue contraction and subsequently restore functionality after the removal of an inhibitory agent. Our results confirm the potential of the platform to study real-time pharmacological effects in contraction-competent 3D SkMOs, showcasing its potential for preclinical drug testing and real-time functional monitoring, adding a new insight into the applicability of microfluidic technology in the field.

## 5. Conclusions

In conclusion, this study demonstrates the feasibility of using 3D skeletal muscle organoids as a physiologically functional model for DMD, as we can recapitulate key aspects of in vitro muscle development using different patient-derived lines. In addition, the small size of our organoids and the minimal number of cells required for each microtissue (fewer than 30,000) may facilitate the generation of a large number of 3D SkMOs, which could be useful in the future for robust drug screening and patient-oriented in vitro studies. Again, a limitation of the present study is the use of a single control cell line, which may limit the representation of inter-individual variability. In future studies, we plan to incorporate multiple control lines to enhance the robustness, reproducibility, and translational relevance of the results. Additionally, our approach corroborates the need to advance in vitro 3D muscle organoids, in light of the state of the field, by properly and correctly expanding these platforms. This would include co-cultures with other cell types (fibroblasts, motoneurons, fibro-adipogenic precursors, etc.) to enhance maturation, allow a more precise structural analysis, and better reproduce the dystrophic environment along with nerve-like stimulation patterns in order to perform more predictable and informative in vitro studies of disease modeling and drug effects.

## Figures and Tables

**Table 1:** Hydrogel composition for each preparation.

	Solution	Final Concentration
Mix A	X µL Cell suspension	
	0.10 µL Aprotinin (25 µg/mL in water)	0.20 µg/mg fibrinogen
	0.06 µL Thrombin (100 U/mL in 0.1% BSA)	0.5 U/mg fibrinogen
Mix B	0.60 µL Geltrex	20% *v*/*v*
	0.60 µL Fibrinogen (20 mg/mL in 0.9% NaCl)	4 mg/mL

**Table 2:** List of gene primers with their unique assay ID.

Gene Symbol	Gene Full Name	Unique Assay ID
MYH2	Myosin, heavy chain 2	qHsaCID0012805
MYH7	Myosin, heavy chain 7	qHsaCID0011217
MYH3	Embryonic myosin heavy chain 3	qHsaCID0015273
MYOG	Myogenin (myogenic factor 4)	qHsaCED0043933
DMD	Dystrophin	qHsaCID0010707
RYR1	Ryanodine receptor 1 (skeletal)	qHsaCID0016836
RPS18	Ribosomal Protein S18 (reference gene)	qHsaCED0037454
UBC	Ubiquitin C (reference gene)	qHsaCED0023867
ITPR3	Inositol 1,4,5-Trisphosphate Receptor Type 3	qHsaCID0020985
ATP2A2	Sarcoplasmic/Endoplasmic Reticulum Calcium ATPase 2	qHsaCID0011088

## Data Availability

All data that support the findings of this study are included within the article (and any Supplementary Files). Further inquiries can be directed to the corresponding author.

## Supplementary Materials

The following supporting information can be downloaded at: https://www.mdpi.com/article/10.3390/biomedicines13051109/s1, Figure S1: Schematic representation of MUSbit-1CTM. Figure S2: Authentic images of custom multi-well plate format for MUSbitTM chips and electrical stimulation device. Figure S3: Authentic images of the microfluidic experimental setup. Figure S4: An authentic image of the comPLATETM interface connected to custom-made electrical stimulation device. Figure S5: Immunofluorescence image of full-length 3D SkMO showing MHC staining. Table S1: Morphological parameters used as acceptance criteria. Video S1a–f: Twitch and tetanic contraction at day 13 for HWT, DMD1, DMD2. Video S2: Calcium transient of HWT tissue at 30 Hz.

## Abbreviations

The following abbreviations are used in this manuscript:

3D SkMO	three-dimensional skeletal muscle organoid
