# Supplementary material for: Patient-Oriented In Vitro Studies in Duchenne Muscular Dystrophy: Validation of a 3D Skeletal Muscle Organoid Platform
Source: Biomedicines. 2025 May 3;13(5):1109. doi: 10.3390/biomedicines13051109 (PMC12109395; doi:10.3390/biomedicines13051109)
Supplement: Supplementary file 1 [file biomedicines-13-01109-s001.zip › Table S1.pptx]

## Slide 1
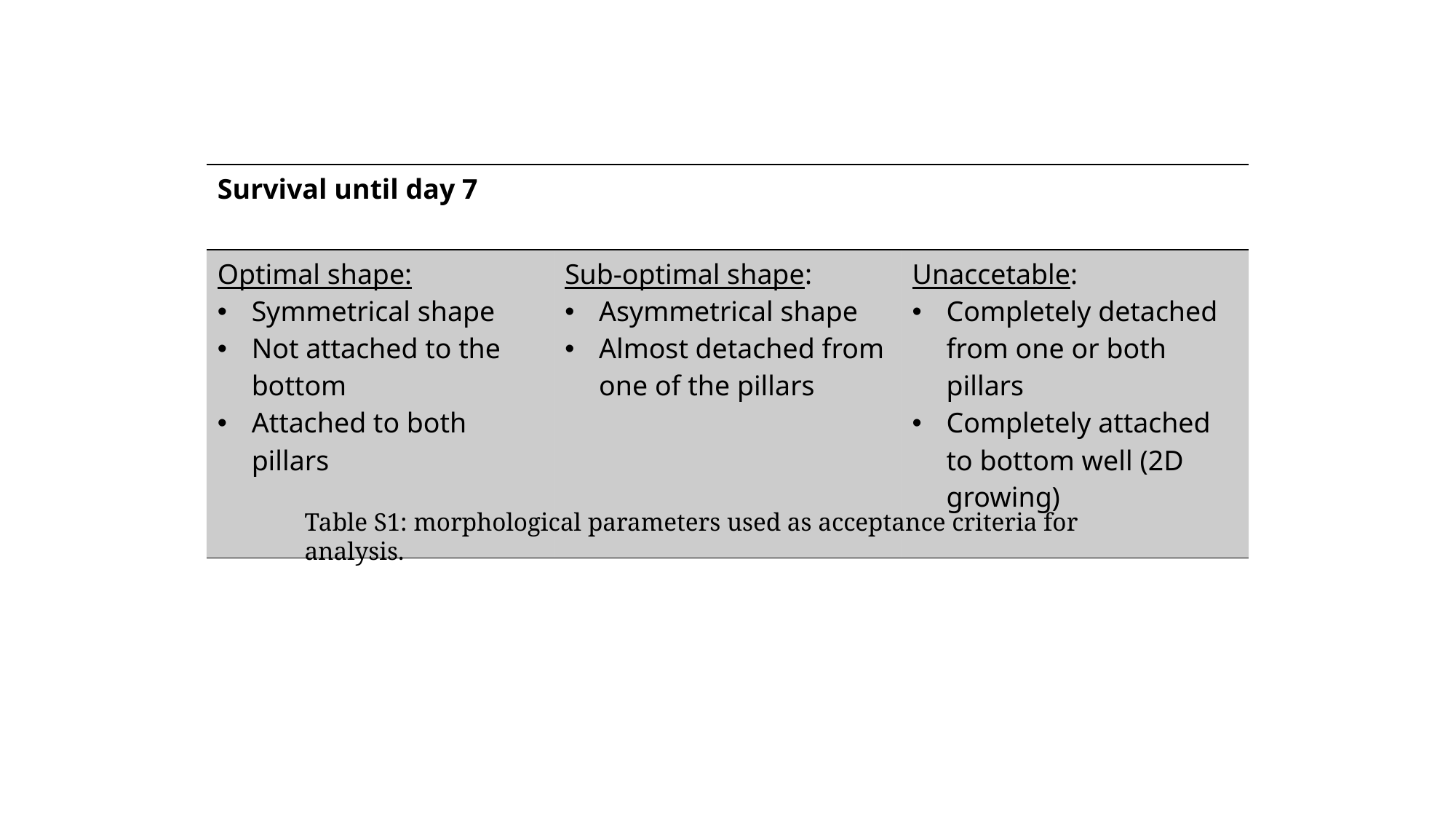

| Survival until day 7 | | |
| --- | --- | --- |
| Optimal shape: Symmetrical shape Not attached to the bottom Attached to both pillars | Sub-optimal shape: Asymmetrical shape Almost detached from one of the pillars | Unaccetable: Completely detached from one or both pillars Completely attached to bottom well (2D growing) |
Table S1: morphological parameters used as acceptance criteria for analysis.
