# Supplementary figures and images for: Patient-Oriented In Vitro Studies in Duchenne Muscular Dystrophy: Validation of a 3D Skeletal Muscle Organoid Platform
Source: Biomedicines. 2025 May 3;13(5):1109. doi: 10.3390/biomedicines13051109 (PMC12109395; doi:10.3390/biomedicines13051109)

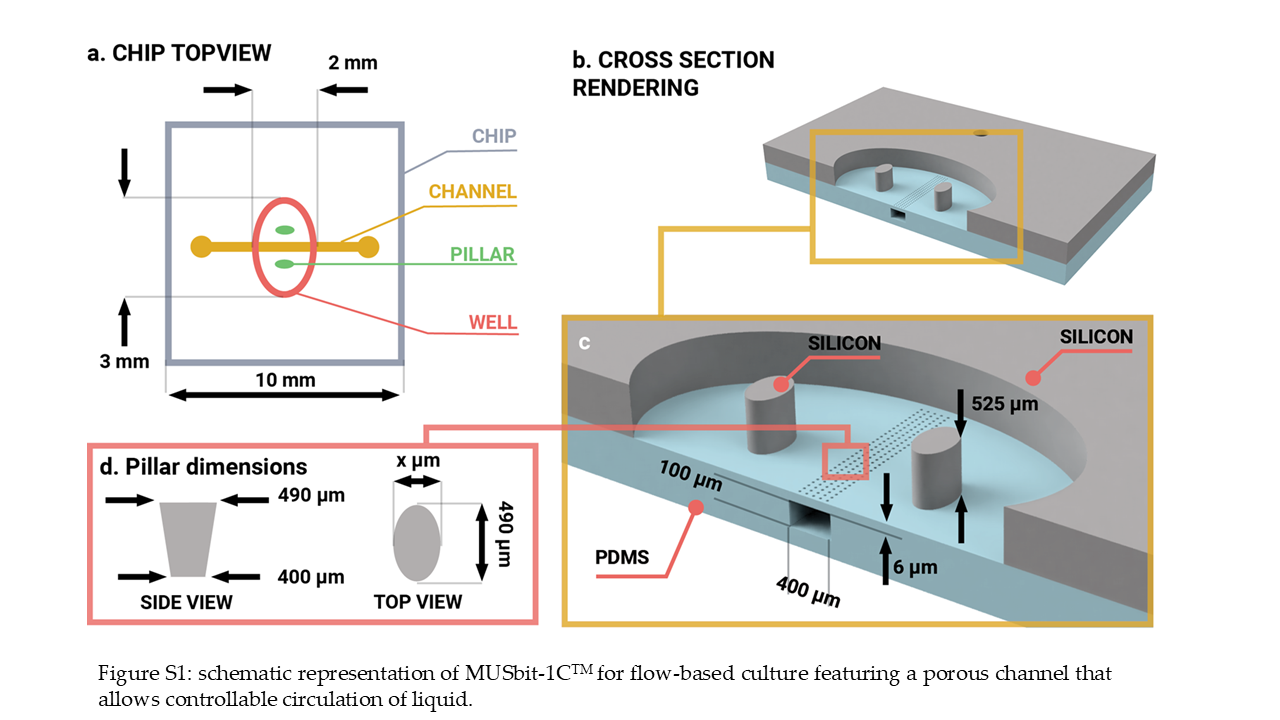

Supplement: Supplementary file 1 [file biomedicines-13-01109-s001.zip › Figure S1.png]

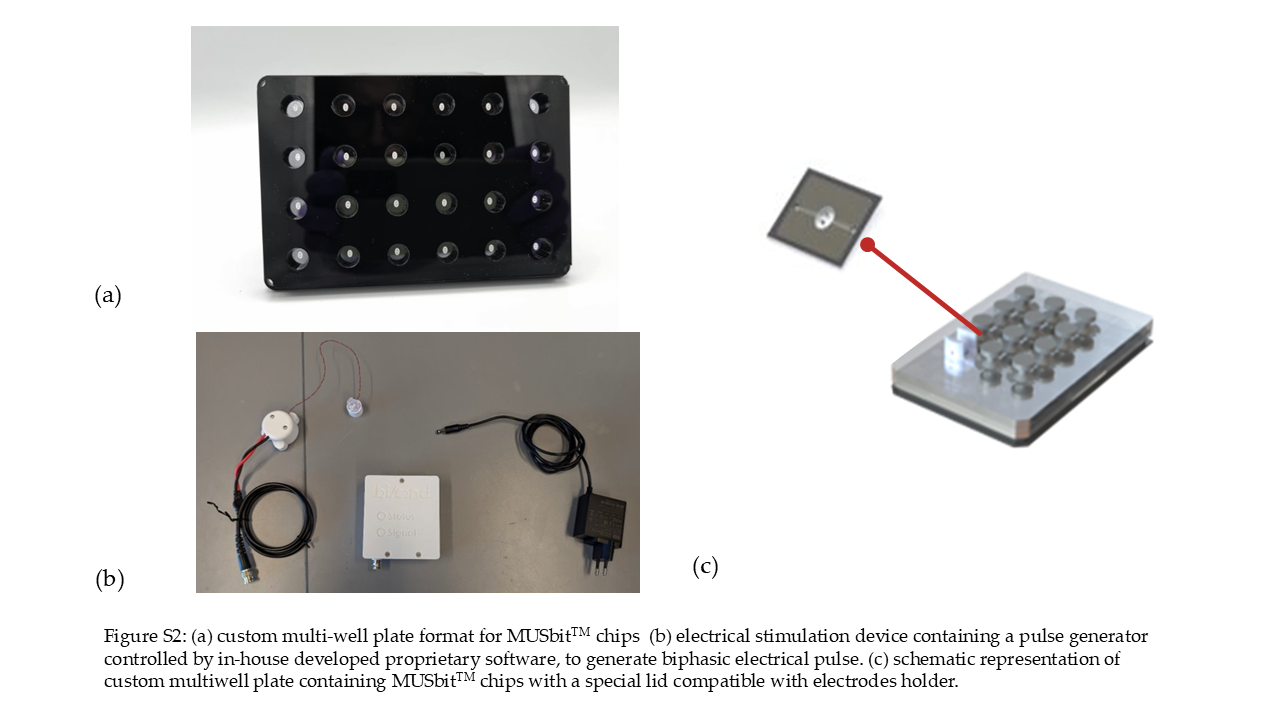

Supplement: Supplementary file 1 [file biomedicines-13-01109-s001.zip › Figure S2.png]

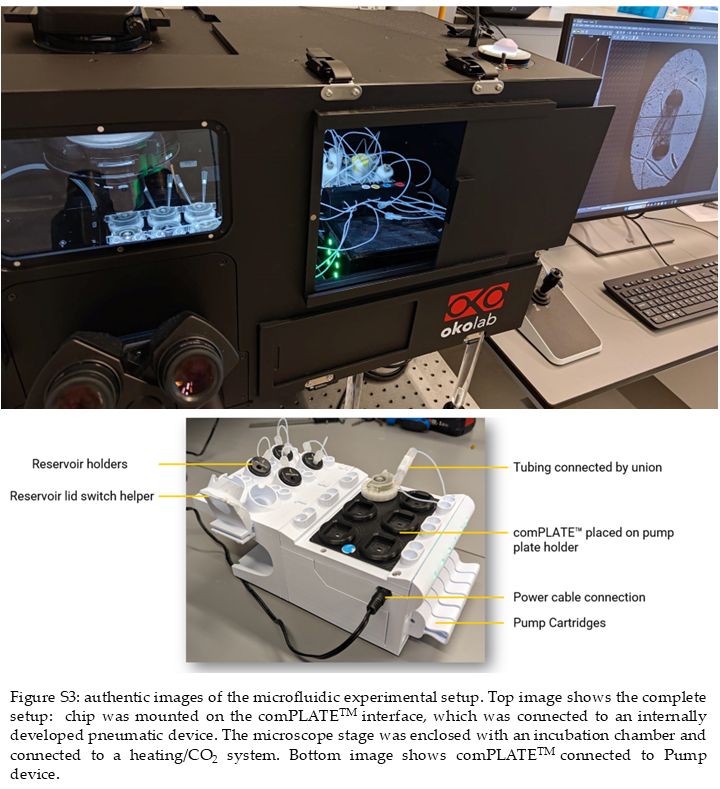

Supplement: Supplementary file 1 [file biomedicines-13-01109-s001.zip › Figure S3.png]

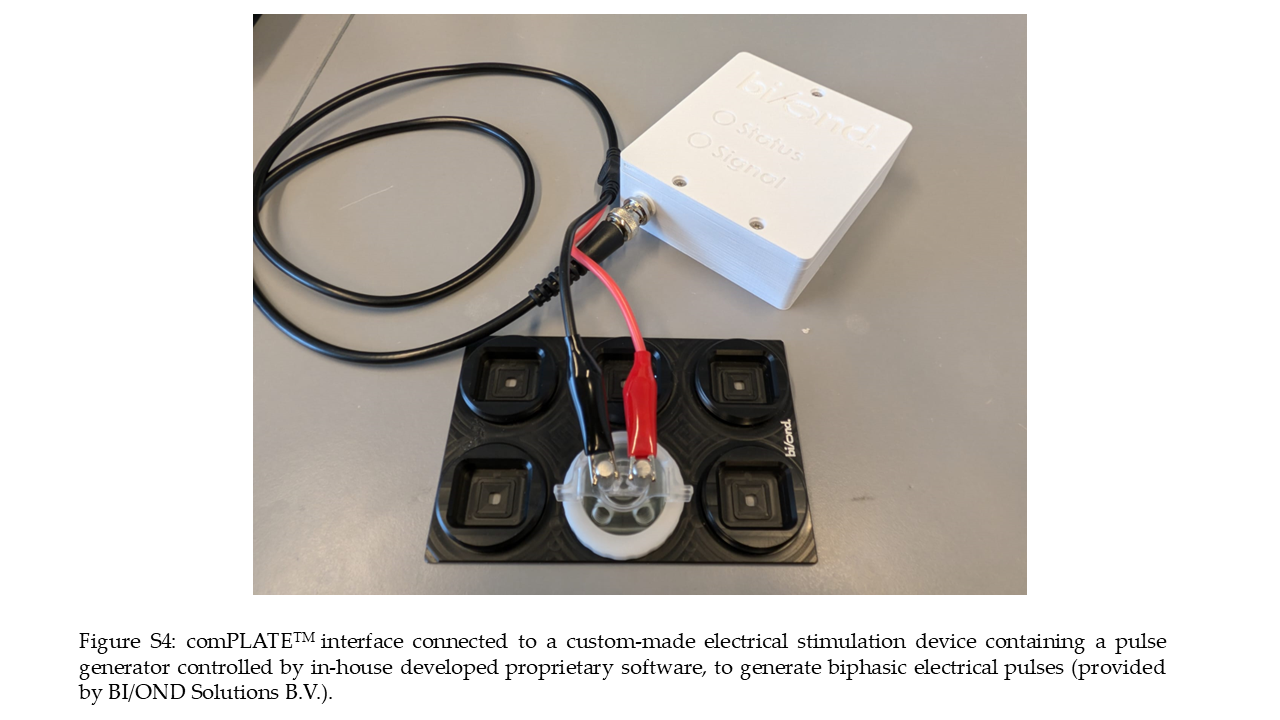

Supplement: Supplementary file 1 [file biomedicines-13-01109-s001.zip › Figure S4.png]

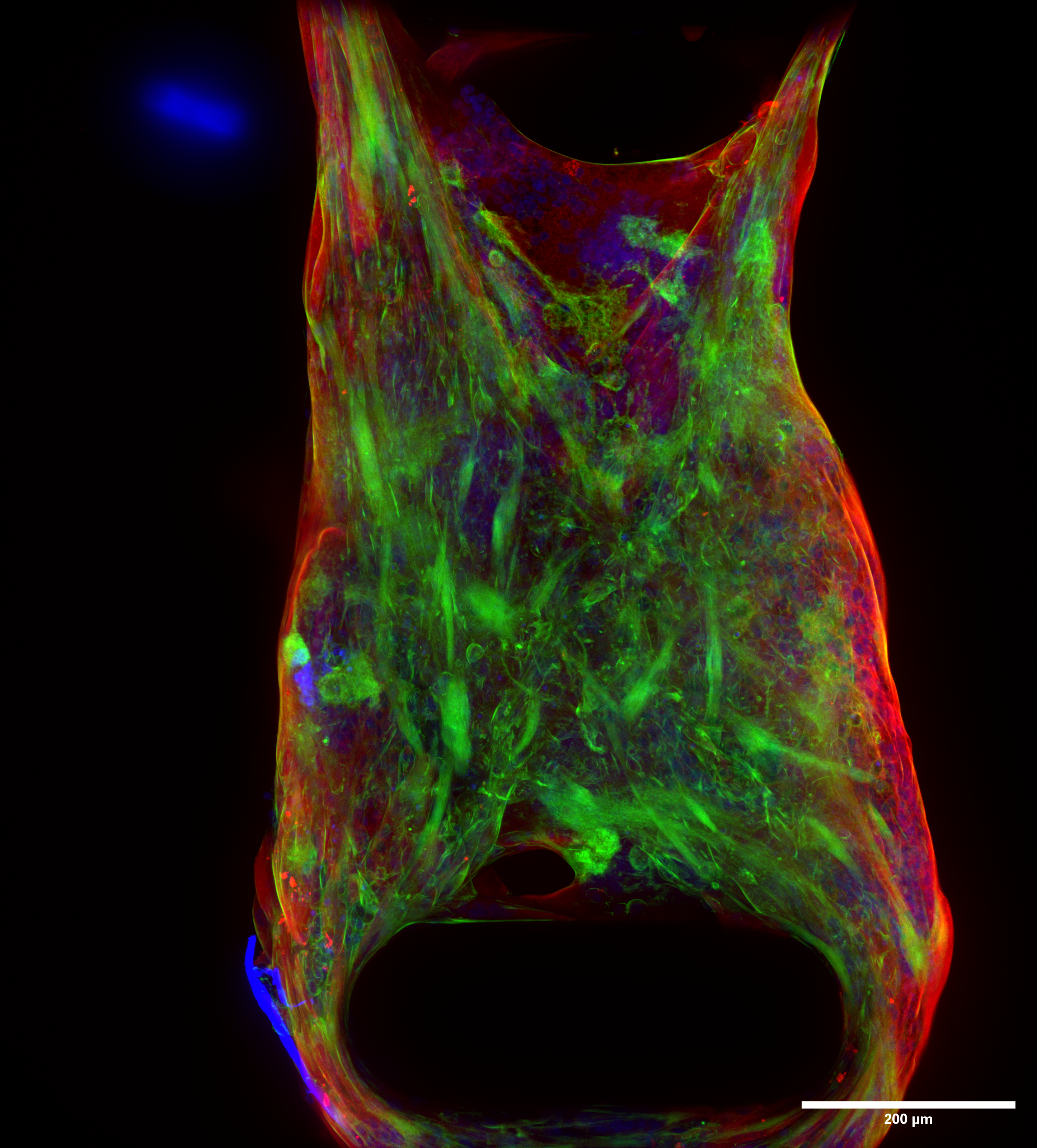

Supplement: Supplementary file 1 [file biomedicines-13-01109-s001.zip › Figure S5.png]

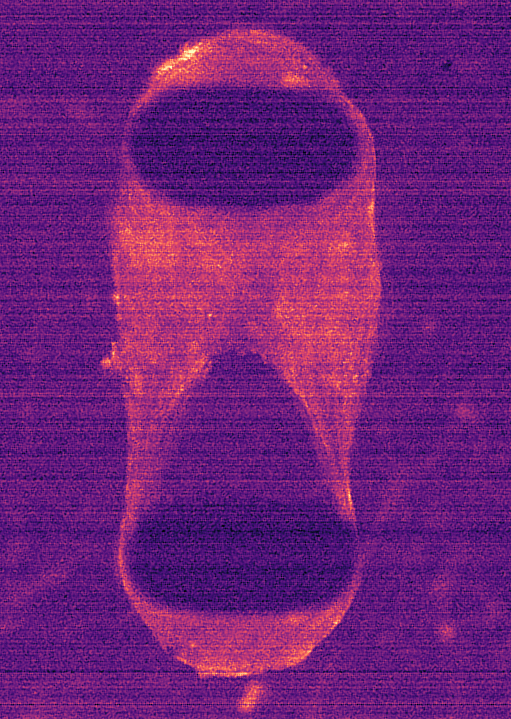

Supplement: Supplementary file 1 [file biomedicines-13-01109-s001.zip › Video S2.gif]
